# De novo assembly and Transcriptome characterization of an endemic species of Vietnam, *Panax vietnamensis* Ha et Grushv., including the development of EST-SSR markers for population genetics

**DOI:** 10.1186/s12870-020-02571-5

**Published:** 2020-07-29

**Authors:** Dinh Duy Vu, Syed Noor Muhammad Shah, Mai Phuong Pham, Van Thang Bui, Minh Tam Nguyen, Thi Phuong Trang Nguyen

**Affiliations:** 1 Vietnam - Russia Tropical Centre, 63 Nguyen Van Huyen, Nghia Do, Cau Giay, Hanoi, Vietnam; 2 Graduate University of Science and Technology (GUST), Vietnam Academy of Science and Technology (VAST), 18 Hoang Quoc Viet, Cau Giay, Hanoi, Vietnam; 3 Department of Experimental Taxonomy & Genetic Diversity, Vietnam National Museum of Nature, Vietnam Academy of Science and Technology (VAST), 18 Hoang Quoc Viet, Cau Giay, Hanoi, Vietnam; 4 Department of Horticulture, Faculty of Agriculture, Gomal University Dera Ismail Khan, Dera Ismail Khan, Pakistan; 5 College of Forestry Biotechnology, Vietnam National University of Forestry, Xuan Mai, Hanoi, Vietnam; 6 Institute of Ecology and Biological Resource, Vietnam Academy of Science and Technology (VAST), 18 Hoang Quoc Viet, , Cau Giay, Hanoi, Vietnam

**Keywords:** Conservation, EST-SSRs, Transcriptome, *Panax vietnamensis*, Population genetics

## Abstract

**Background:**

Understanding the genetic diversity in endangered species that occur inforest remnants is necessary to establish efficient strategies for the species conservation, restoration and management. *Panax vietnamensis* Ha et Grushv. is medicinally important, endemic and endangered species of Vietnam. However, genetic diversity and structure of population are unknown due to lack of efficient molecular markers.

**Results:**

In this study, we employed Illumina HiSeq™ 4000 sequencing to analyze the transcriptomes of *P. vietnamensis* (roots, leaves and stems). Raw reads total of 23,741,783 was obtained and then assembled, from which the generated unigenes were 89,271 (average length = 598.3191 nt). The 31,686 unigenes were annotated in different databases i.e. Gene Ontology, Kyoto Encyclopedia of Genes and Genomes, Nucleotide Collection (NR/NT) and Swiss-Prot for functional annotation. Further, 11,343 EST-SSRs were detected. From 7774 primer pairs, 101 were selected for polymorphism validation, in which; 20 primer pairs were successfully amplified to DNA fragments and significant amounts of polymorphism was observed within population. The nine polymorphic microsatellite loci were used for population structure and diversity analyses. The obtained results revealed high levels of genetic diversity in populations, the average observed and expected heterozygosity were H_O_ = 0.422 and H_E_ = 0.479, respectively. During the Bottleneck analysis using TPM and SMM models (*p* < 0.01) shows that targeted population is significantly heterozygote deficient. This suggests sign of the bottleneck in all populations. Genetic differentiation between populations was moderate (*F*_*ST*_ = 0.133) and indicating slightly high level of gene flow (*Nm* = 1.63). Analysis of molecular variance (AMOVA) showed 63.17% of variation within individuals and 12.45% among populations. Our results shows two genetic clusters related to geographical distances.

**Conclusion:**

Our study will assist conservators in future conservation management, breeding, production and habitats restoration of the species.

## Background

*Panax* species (Araliaceae) are medicinally important plants of North America and eastern Asia [1, 2]. In 19 species of the *Panax* genus [3, 4], three known species, *Panax vietnamensis*, *P. stipuleanatus* and *P. bipinatifidus* are related to the high mountains of Vietnam [5, 6]. *Panax* species are characterized by the presence of ginsenosides, which refer to a series of dammarane [7]. *P. vietnamensis* was found for the first time in Ngoc Linh mountain of Kon Tum province [8]. *P. vietnamensis* (Vietnamese Ginseng) is an endemic species of Vietnam, rich in saponin compound [1, 8, 9]. It is a perennial plant and grows up to 1 m in height, with a diameter of 4–8 mm under humus forest canopy. It has oval-shaped leaves with a serrated margin. The flowers are an inflorescence and fruit turn red at maturity with 1–2 white color seeds. *P. vietnamensis* is routinely used for the treatment of many serious diseases and enhancement of body stamina during mountainous journeys by the Sedang ethnic group [1]. Excessive exploitation in the past few decades and slow growth rate, poor regeneration of *P. vietnamensis,* the natural population sharply declines and put the species in endangered [10]. Therefore, it is listed in in the Vietnam Red Data Book 2007 (EN A1a,c,d, B1 + 2b,c,e) [11]. It is currently in the protection list of both the central and local governments of Vietnam. It needs urgent conservation and restoration, but main hurdle is the unexplored structure and genetic diversity of the *P. vietnamensis* wild populations due to unavailability of informative and reliable molecular markers for *P. vietnamensis.*

Simple sequence repeats (SSRs) markers are useful tools for research in plant genetics, breeding, identification of individuals, species and varieties, and to generate genetic maps because of the allelic sequence diversity as they are widely spread in the genome and have high levels of the polymorphism, co-dominant inheritance, abundance, maximum reproducibility, multi-allelic variation, and good genome coverage [12–18]. The expressed sequence tags (EST) availability, enhanced SSR identification possibility in some woody plants [19]. As a functional molecular marker, SSRs generated from expressed sequence tags (EST-SSRs) can investigate the effects of environmental heterogeneity and local adaptation due to its tight linkage with functional genes controlling phenotype [20–22]. Up till now numbers of EST-SSRs developed and checked for polymorphism in many species, such as sweet potato [23], *Sesamum indicum* [24], radish [25], *Cymbidium sinense* [26], Chinese bayberry [27], Silver fir [28], *Salix*, *Populus*, *Eucalyptus* [29]; *Rosa roxburghii* [30], *Neottopteris nidus* [16], Lacquar tree [31], Bread wheat [32], Proso Millet [33], Almond [34] and Ginseng [35]. Limited genomic resources have been developed for *Panax* species so far, e.g., *P. vietnamensis* var. *fuscidiscus* [2], *P. ginseng* [35, 36] as compared to other crop.

Illumina HiSeq™ 4000 is a new-generation method to permit a comprehensive analysis in the gene expression profile, have provided fascinating opportunities in life sciences and facilitated transcriptomes sequencing at low-cost and rapid identification of EST-SSRs [16, 22, 25, 26, 34, 35, 37–39]. Transcriptomes de novo assembly is indispensable for functional genomics or markers mining in non-model plants study, especially when genome sequence is not available [16, 23, 30, 37–39]. Up till now, only nucleotide sequences of *P. vietnamensis* var. *fuscidiscus* and *P. ginseng* in the *National Center for Biotechnology Information (*NCBI) database are available (August 2019), while no ESTs are available in GenBank for *P. vietnamensis*. Previous studies investigated the genetic variation and verified the taxonomic status of the *Panax* species at the molecular level [40–49]. However, few researchers studied the *P. vietnamensis* in Vietnam [50–52].

In the current study, (i) we have produced global transcriptome from *P. vietnamensis* using the Illumina HiSeq™ 4000 and analyzed functions, classification and metabolic pathways of the resulting transcripts. (ii) Then we have developed a set of EST-SSRs for *P. vietnamensis* and (iii) confirmed the efficacy of these markers by studying the genetic structure and diversity of three wild populations of *P. vietnamensis*. (iv) At last, the influences of geographical distance on genes flow within wild population were tested.

## Results

### De novo assembly and Illumina sequencing of *P. vietnamensis* transcriptomes

**Fig. 1:**
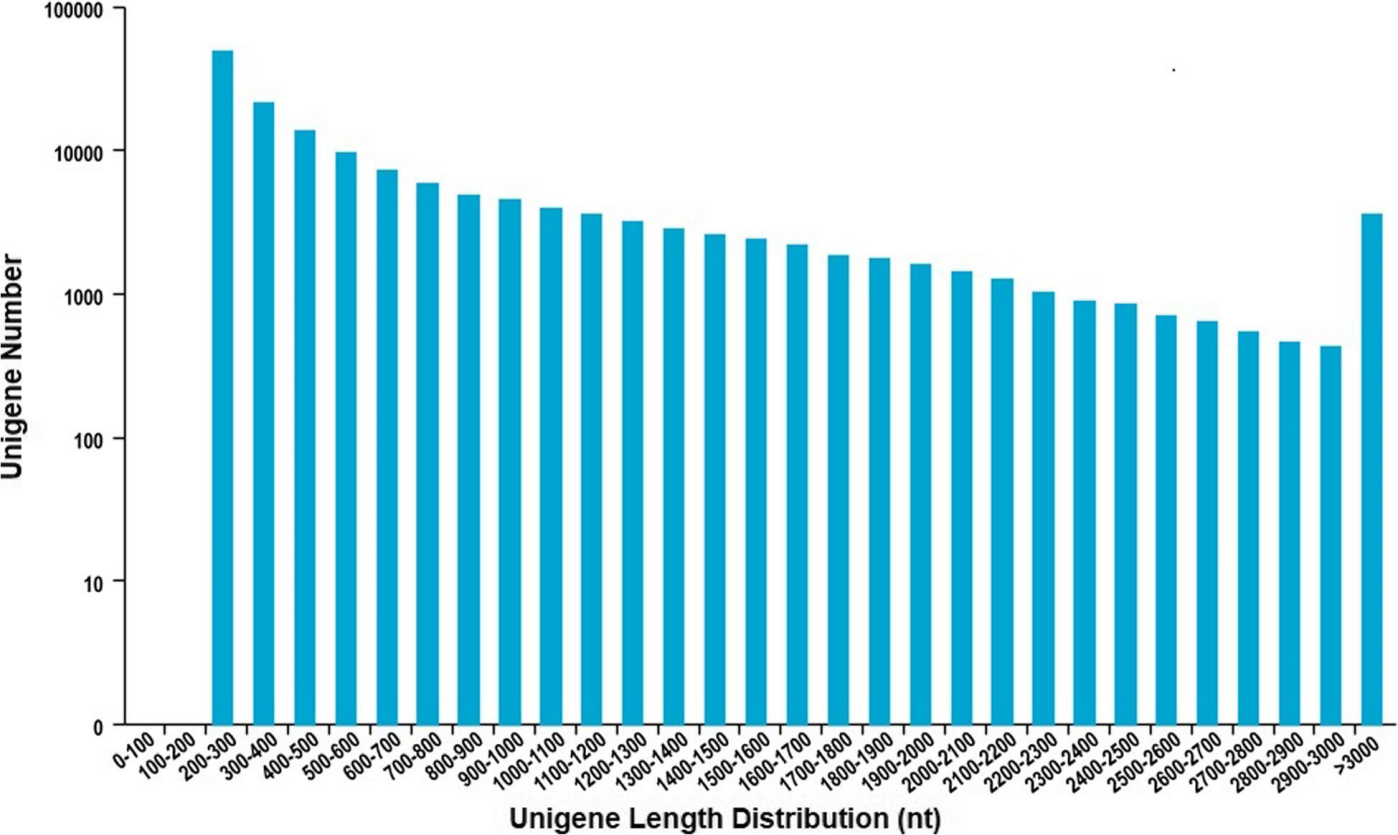
Distribution of unigenes lengths resulting from de novo transcriptome assembly of *P. vietnamensis*

Transcriptome sequencing of *P. vietnamensis,* a total of 7,083,775,547 bases were generated and after a stringent quality check 23,741,783 paired-end, high quality, clean reads were obtained with 97.52% Q20 and 93.5% Q30 bases, while the GC contents were 51.43%. De novo assembly was further checked through Trinity and as a result, 153,074 transcripts with 117,954,630 bp were detected, while N50 value was1,268 bp with an average length of 770.572 bp. Among total number of transcripts, 48,314 (31.56%) transcripts were between 200 and 300 bp; 35,174 (22.98%) transcripts ranged from 301 to 500 bp; 32,031 (20.93%) transcripts ranged from 501 to 1000 bp; 25,800 (16.85%) transcripts ranged from 1001 to 2000 bp and 11,755 (7.68%) transcripts were larger than 2000 bp. Meanwhile, the assembly produced 89,271 unigenes having a N50 length of 942 bp (average length = 598.319 bp) were assembled and retained for analyses. In these unigenes 39,947 (44.75%) were between 200 and 300 bp; 22,049 (24.70%) ranged from 301 to 500 bp; 13,669 (15.31%) ranged from 501 to 1000 bp; 9048 (10.14%) ranged from 1001 to 2000 bp and 4558 (5.11%) were larger than 2000 bp (Fig. 1, Table 1).

**Table 1:** Overview of de novo sequence assembly for *P. vietnamensis*

Length range (bp)	Unigene	Transcripts
200–300	39,947 (44.75%)	48,314 (31.56%)
300–500	22,049 (24.70%)	35,174 (22.98%)
500–1000	13,669 (15.31%)	32,031 (20.93%)
1000–2000	9048 (10.14%)	25,800 (16.85%)
> 2000	4558 (5.11%)	11,755 (7.68%)
Total Number	89,271	153,074
Total Length	53,412,541	117,954,630
N50 Length	942	1268
Mean Length	598.3191	770.5725989

### Functional annotation of assembled unigenes

**Fig. 2:**
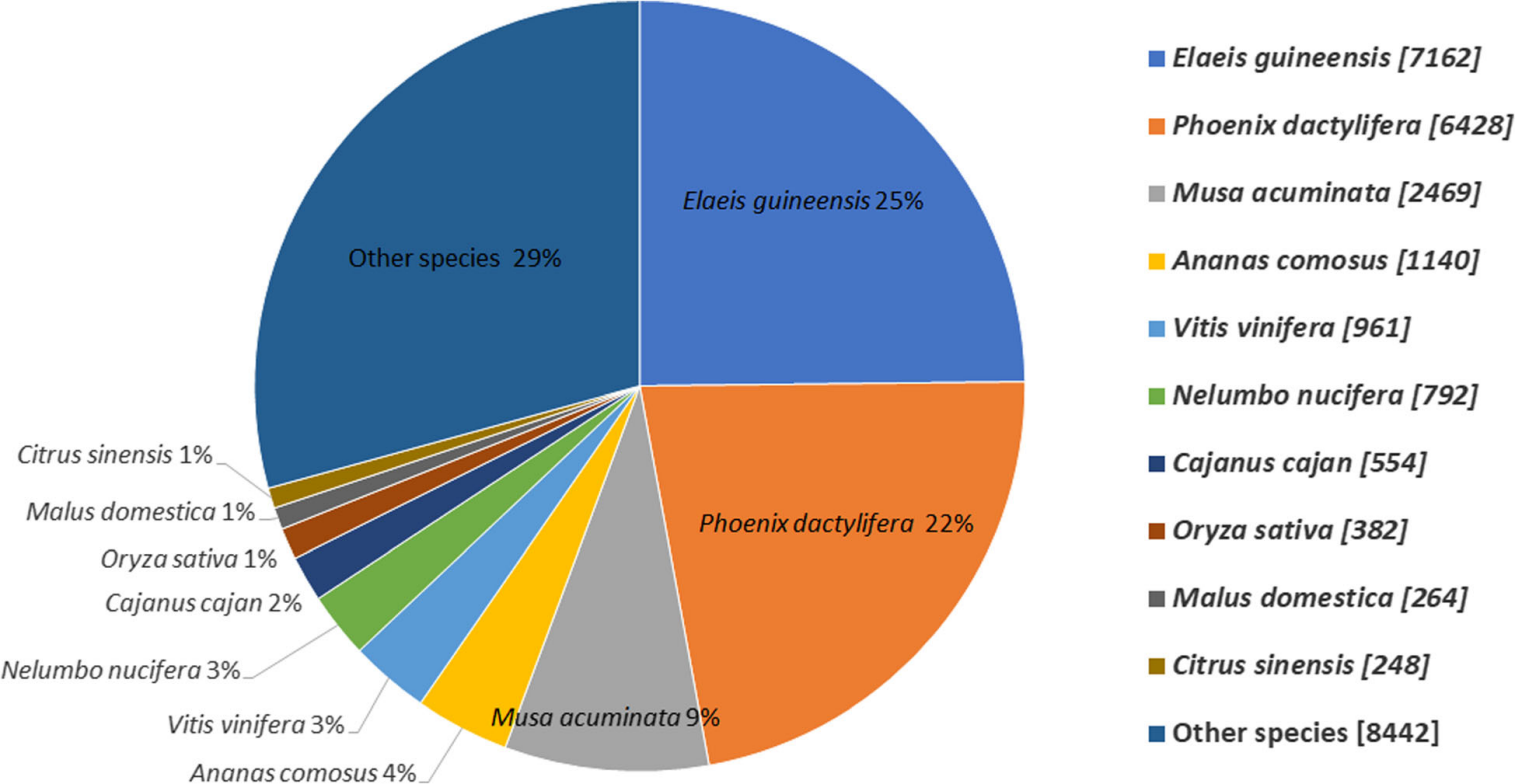
Distribution of species search of unigenes against the *Nr* database

For functional annotation analyses the unigenes were blasted against the seven databases (COG, GO, KEGG, KOG, Pfam, Swissprot and NR), a total 31,686 matched sequences was found (Table 2). Among the 89,271 unigenes, resulted successful annotation of 7647 (8.57%) in the *COG* databases, 14,568 (16.32%) in the *GO* database, 5838 (5.42%) in *KEGG* database, 16,860 (18.89%) in *KOG* database, 18,600 (20.845) in Pfam, 19, 228 (21.54%) in the Swiss-Prot protein database and 16,659 (18.66%) unigenes in the NR protein database (Table 2). For the species distribution *BLASTx* was used to search against Nr databases, the *P. vietnamensis* transcriptome shows highest similarities with *Elaeis guineensis* (25%) followed by *Phoenix dactylifera* (22%) and *Musa acuminata* (9%) (Fig. 2).

**Table 2:** Functional annotation of *P. vietnamensis* in different databases

Annotated database	Annotated_No.	Percentage (%)	300–1000 (bp)	≥ 1000(bp)
COG	7647	8.57	1905	4695
GO	14,568	16.32	5097	6695
KEGG	5838	5.42	1876	3142
KOG	16,860	18.89	6059	7636
Pfam	18,600	20.84	6061	10,038
Swissprot	19,228	21.54	7150	9213
NR	16,659	18.66	11,122	11,915
All	31,686	35.49	12,160	12,052

**Fig. 3:**
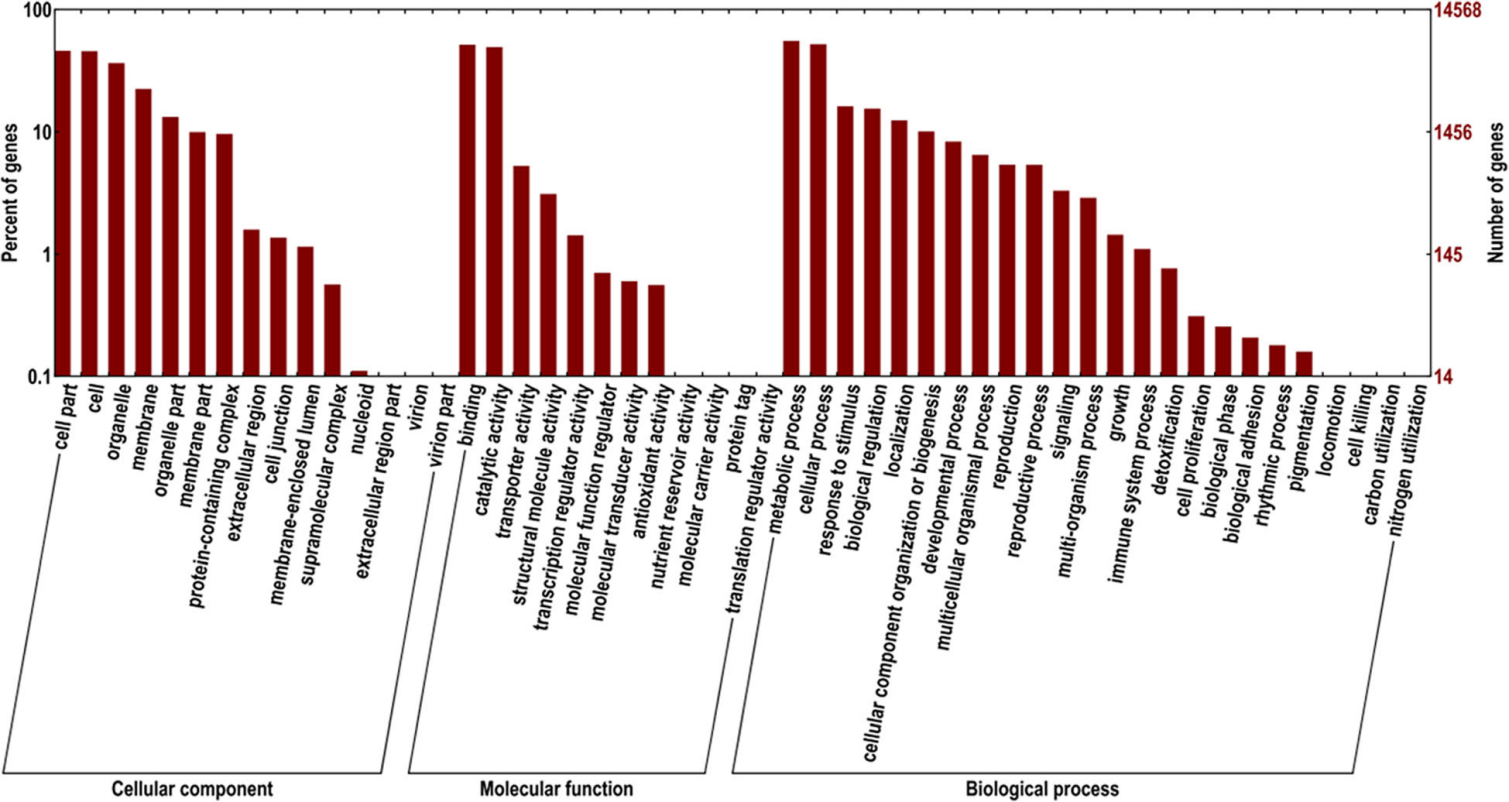
Gene Ontology classification of unigenes

Based on *Nr* annotations, we used the *GO* system to categorize the possible functions of the unigenes. A total of 72,183 (80.86%) unigenes was successfully grouped into three classes (biological process, molecular function and cellular component) and 51 subclasses (Fig. 3). The biological process was the top category (28,653; 39.69%), while subcategories were “metabolic process” (8016; 27.98%) “cellular process” (7528; 26.27%) and “response to stimulus” (2347; 8.19%). The cellular component unigenes were 27,232 (37.72%), classified into “cell part” (6645; 24.40%) “cell” (6596; 24.22%) and “organelle” (5269; 19,35%). The 16,298 (22.58%) unigenes were related to “molecular function” in which prominent subcategories are “binding” (7459; 45.77%) and “catalytic activity” (7130; 43.75%). It was also observed that the few genes are enriched in the terms of “nutrient reservoir activity”, “molecular carrier activity”, “protein tag” and “translation regulator activity”.

**Fig. 4:**
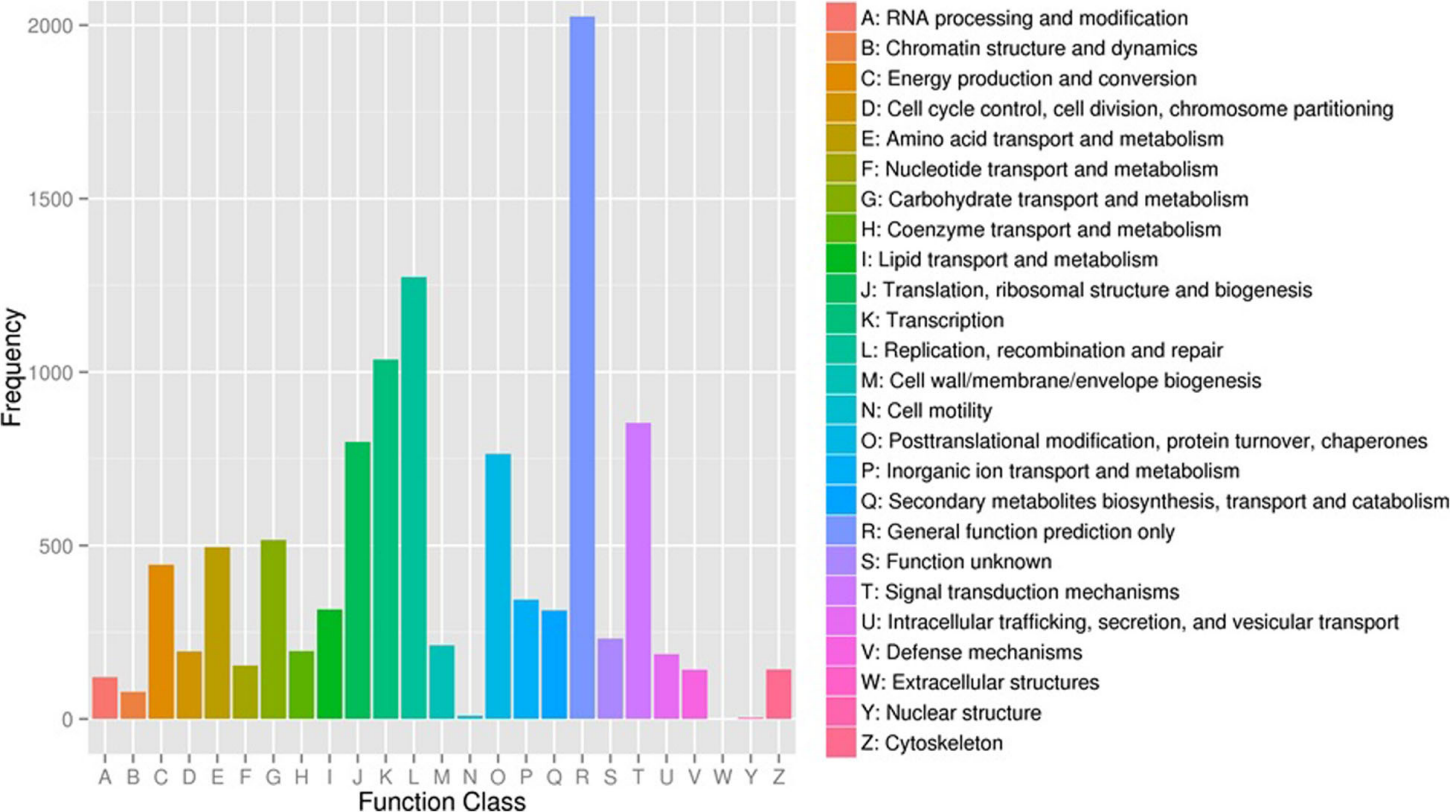
Clusters of orthologous groups (*COG*) classification

A total of 7647 unigenes were assigned to Clusters of Orthologous Groups (COG), to check the reliability of the transcriptome library and effectiveness of the annotation process, for functional prediction and classification (Fig. 4). COG-annotated putative proteins were functionally classified into 25 categories. The top groups were “general function prediction only” (9089), “translation, ribosomal structure and biogenesis” (3388) and “transcription” (977), respectively. However, only few unigenes were annotated as “extracellular structures” and “nuclear structure.”

**Fig. 5:**
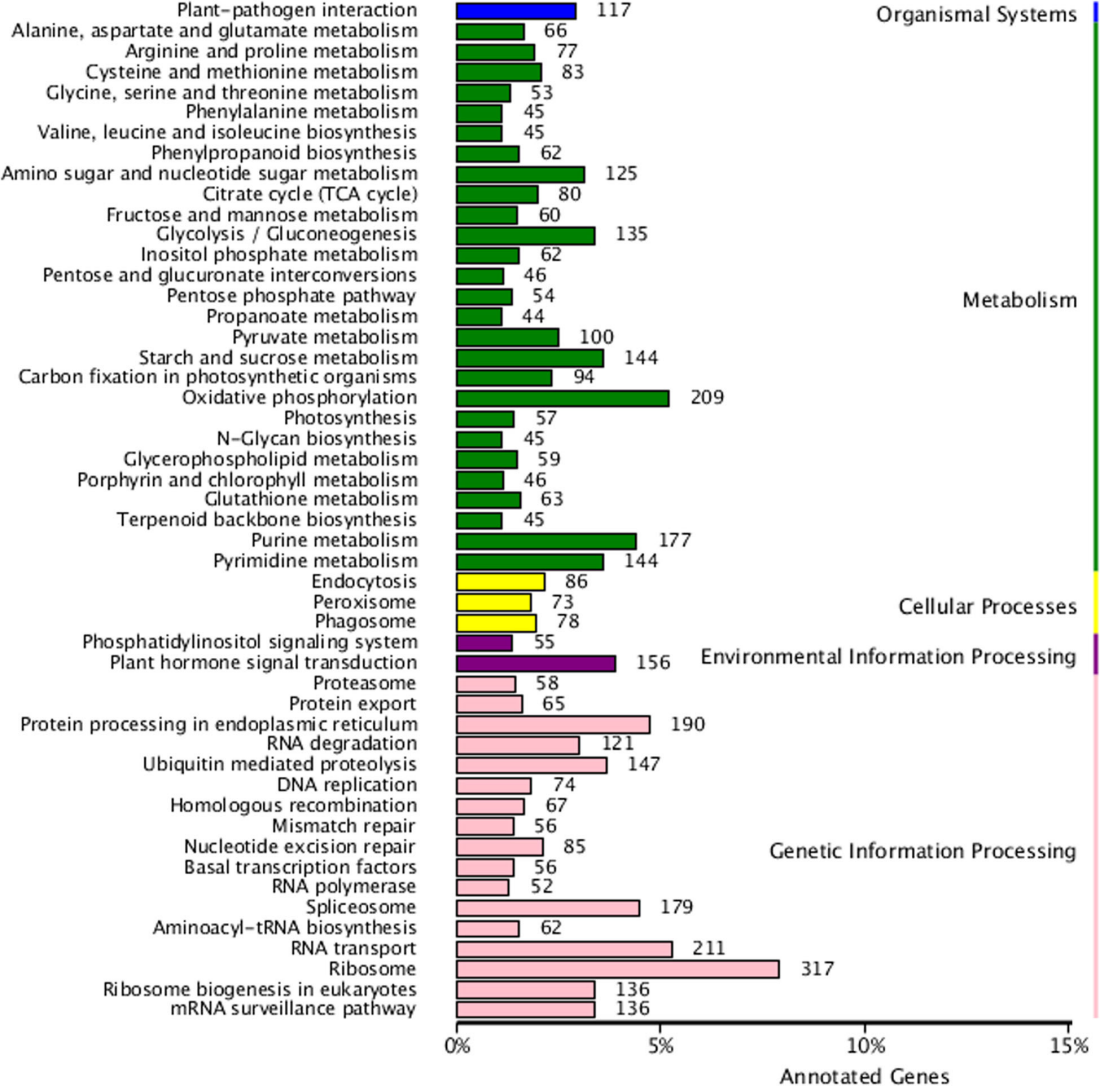
Clusters of orthologous groups KEGG classification

The KEGG pathway analysis was used to explore the biological pathways in *P. vietnamensis* that might be active with an E value cutoff < 10^− 5^. The 5838 unigenes was significantly matched in the *KEGG* database and assigned to 118 *KEGG* functional pathways (Fig. 5). The specific pathways, including plant hormone signal transduction, purine metabolism, ribosome, RNA transport spliceosome and many more pathways. In addition, 45 unigenes were in the terpenoid backbone biosynthesis pathway.

### EST-SSR markers development and characterization from the *P. vietnamensis* transcriptome

To develop new molecular markers and to check the assembly quality, the 89,271 unigenes were used for microsatellites mining that were well-defined as di- to hexa-nucleotide motifs. The SSRIT was used and identified 11,343 EST-SSRs. The 6949 sequences contained one SSR, while 2763 sequences have more than one SSR. The EST-SSRs frequency was 12.71%, and one EST-SSRs distribution density was 5.98 kilobases (kb) in the unigenes.

**Fig. 6:**
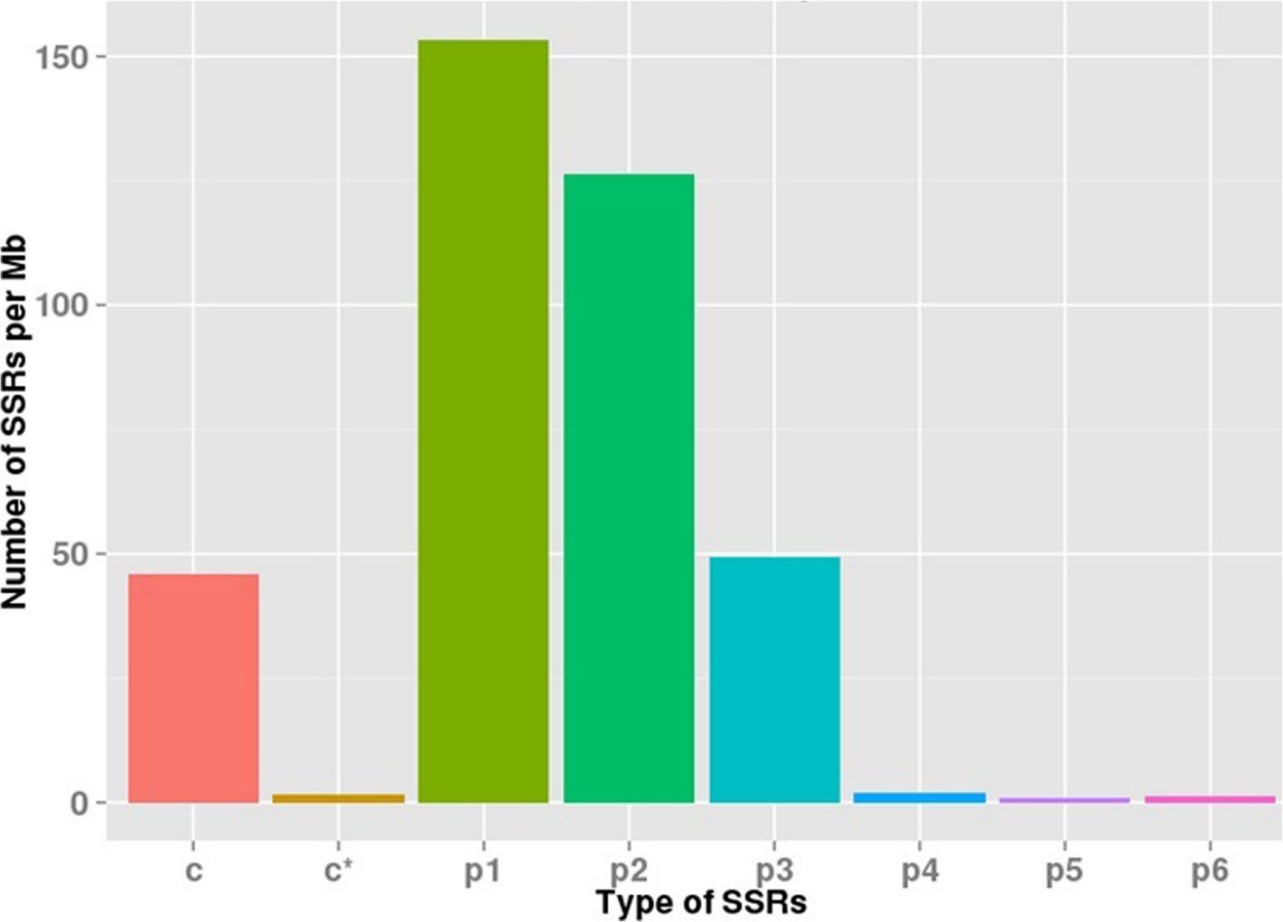
Distribution type of EST-SSRs of *P. vietnamensis*

**Fig. 7:**
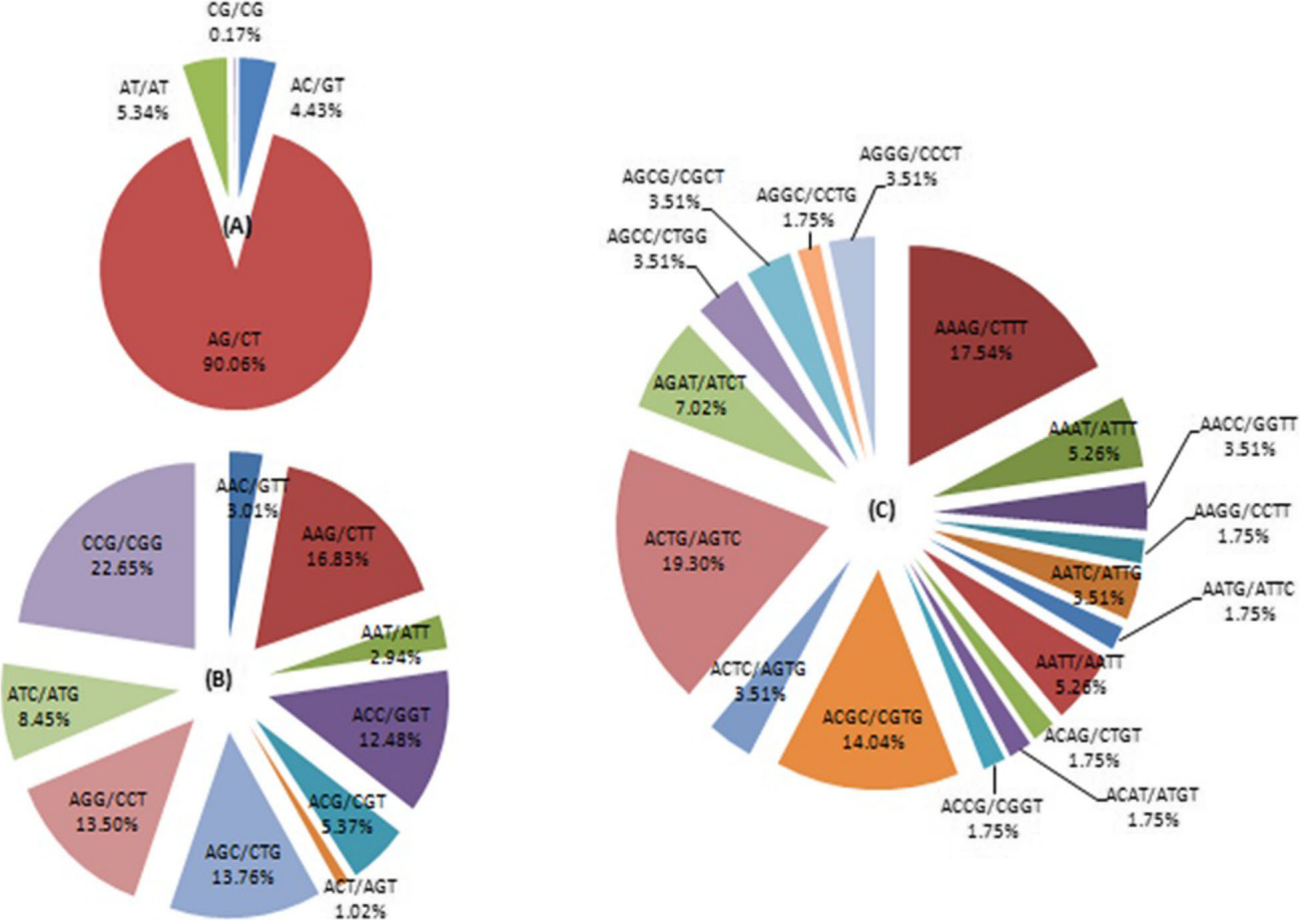
Percentage of different motifs in di-nucleotide (**a**), tri-nucleotide (**b**), and tetra-nucleotide (**c**) repeats in *P. vietnamensis*

The potential EST-SSRs were analyzed for frequency, type, and distribution. The most common repeat motif was mono-nucleotide (5004; 44.12%), followed by di-nucleotide (4648; 40.98%), tri-nucleotide (1563; 13.78%), tetra-nucleotide (66; 0.58%), hexa-nucleotide (29; 0.26%), and penta-nucleotide (32; 0.28%) repeats (Fig. 6; Table 3). EST-SSRs with ten repeat motifs (2040; 20.50%), six repeat motifs (1363; 12.02%), five repeat motifs (925; 8.15%), seven repeat motifs (862; 7.6%), eight repeat motifs (594; 5.24%), and nine repeat motifs (428; 3.77%) were the most common, respectively. The dominant motif in di-nucleotide repeats was AG/TC (90.06%), followed by AT/TA (5.34%) and AC/TG (4.43%). In type 10 of tri-nucleotide repeats, the highest motif distribution was CCG/GGC (22.65%), while the common motif in tetra-nucleotide repeats was ACTG/TGAC (19.30%) (Fig. 7). Additionally, 16 and 17 different types of penta and hexa-nucleotides repeats of EST-SSRs were detected, respectively.

**Table 3:** The distribution of EST-SSRs based on the number of repeat units

Number of repeat units	Mono-	Di-	Tri-	Tetra-	Penta-	Hexa-	Total	Percentage (%)
5	0	0	851	34	20	20	925	8.15
6	0	965	363	24	2	9	1363	12.02
7	0	705	147	2	5	3	862	7.60
8	0	485	105	3	1	0	594	5.24
9	0	394	32	2	0	0	428	3.77
10	2040	260	24	1	0	0	2325	20.50
> 10	2964	1839	41	1	1	0	4846	42.72
Total	5004	4648	1563	66	29	32	11,343	100
Percentage (%)	44.12	40.98	13.78	0.58	0.26	0.28	100

### Genetic structure and diversity of population

A total of 98 individuals from three *P. vietnamensis* populations produced 27 different alleles, ranging from 120 to 265 bp at the nine loci (Table 4). In the current study, the polymorphism information content (PIC) value ranged from 0.325 (L111) to 0.493 (L145), with an average of 0.361. The number of detected alleles per locus (A) in overall 98 individuals ranged from two at two loci (L119 and L145) to four at two loci (L37 and L111) with an average value of three. The lowest detected heterozygosity (H_O_) was found at locus L73 (0.178) and the highest at locus L111, with an average of 0.422. Similarly, the lowest expected heterozygosity (H_E_) was recorded for locus L73 (0.208) and the highest for locus L37 (0.65), with an average of 0.479. The value of fixation index (F) in overall population for each locus, average 0.14, ranging from − 0.185 (L111) to 0.386 (L115).

**Table 4:** Characterization and polymorphism levels of nine microsatellite loci in *P. vietnamensis*

Primers	Primer sequence (5′–3′)	Repeat motif	Fragment size (bp)	Ta (°C)	***A***	***PIC***	***Ho***	***He***	***F***	GenBank accession no
L37	F: GAGCGGGAGGGAGAGAGA R: CTTTCTCGTCGTCGTCATCA	(CATCAC)7	120–180	55	4	0.354	0.629	0.650	0.033	MK802095
L39	F: TTTGCCTCACTCCCCTGTAG R: AGAAGGAGGAGAGACCGAGG	(AGCGGC)5	176–201	55	3	0.332	0.439	0.511	0.146	MK802096
L73	F: TCTTGGGGATTGTGAAGGAG R: TTAAGGAACAGTGGCAGCAG	(TCTA)8	205–225	55	3	0.394	0.178	0.208	0.146	MK802097
L111	F: GCTCCACAACTCACTCCTCC R: TCTGTTCAGCTTCGTCCTCC	(TTC)11	197–230	55	4	0.325	0.723	0.610	−0.185	MK802098
L115	F: CCCCATCATTCCATTGGTAG R: CTCAATCCCATCACGAGGAC	(TGT)10	221–239	55	3	0.442	0.386	0.628	0.386	MK802099
L119	F: CGTGTGTTACTGTTGTGGGG R: CGATTCTCACTCCCACCATT	(TGA)10	148–166	55	2	0.471	0.429	0.439	0.024	MK802100
L139	F: AATCATGTGGGACCGAAGAG R: TTGCATTTGGTTTTCTGTGC	(GAA)18	198–249	55	3	0.442	0.217	0.366	0.408	MK802101
L145	F: CCGTCTCCTTCAACTGCTTC R: AGTTGGGAATGAAGATTGCG	(CTT)15	247–265	55	2	0.493	0.231	0.371	0.379	MK802102
L149	F: CCTCCCCAAATCCTCCTCTA R: GACCTCTCCAGCTCCAACAG	(CTC)10	164–221	55	3	0.369	0.569	0.529	0.076	MK802103

*N*ote: *The number of alleles per locus (A), Observed heterozygosities (H*_*O*_*), expected heterozygosities (H*_*E*_*), the fixation index (F)*

At population level, the values of genetic diversity are shown in Table 5, including the alleles mean number (A = 2.6), the effective alleles numbers (A_E_ = 2.2), the proportion of polymorphic loci (92.59%), the observed heterozygosity (H_O_ = 0.422) and expected heterozygosity (H_E_ = 0.479). The fixation index (F) was positive for all the populations (F = 0.13). Therefore, these results showed heterozygosity deficiency and significant inbreeding (*p* < 0.05). Seven loci of the nine loci had positive fixation and indicating high homozygosity and inbreeding. However, among the loci, five loci had significant inbreeding (*p* < 0.05). Two loci had negative values.

**Table 5:** Genetic diversity within *P. vietnamensis* populations at nine loci

Populations	N	A	A_**E**_	P%	H_**O**_	H_**E**_	F	***P*** value of bottleneck	
								TPM	SMM
**DT**	32	2.6	2.1	88.89	0.412	0.454	0.114*	0.002	0.004
**TN**	18	2.6	2.1	88.89	0.444	0.473	0.092	0.002	0.002
**KT**	48	2.8	2.2	100.0	0.410	0.510	0.185*	0.001	0.001
**Mean**		2.6	2.2	92.59	0.422	0.479	0.130*		

Note: *N = population size; A = mean number of alleles per locus; A*_*E*_ = *mean number of effective alleles; P% = percentage of polymorphic loci; H*_*O*_*and H*_*E*_ = *mean observed and expected heterozygosities, respectively; F* = fixation index with *p < 0.05

During the Bottleneck analysis using Stepwise mutation model (SMM) and two phase model (TPM) models (*p* < 0.01) shows that targeted population is significantly heterozygote deficient (Table 5). This suggests sign of the bottleneck in all population.

The analysis of molecular variance (AMOVA) showed that total variation was highly significant (*p* < 0.001) within individuals i.e. 63.17% (Table 6). The *F*_*ST*_ were significant (*p* < 0.05), values range was from 0.072 to 0.182 (average = 0.133) and with 1.63 gene flow. Low genetic differentiation value (*F*_*ST*_ = 0.072) was found between DT and TN population, while high value (*F*_*ST*_ = 0.182) was between DT and KT population (Table 7).

**Table 6:** Analysis of molecular variance in *P. vietnamensis* from three populations

Source of variation	df	Sum of squares	Variance components	Total variation (%)	***P*** value
Among populations	2	76.513	0.723	24.38	
Among individuals within populations	96	208.939	0.369	12.45	
Within individuals	98	155.500	1.873	63.17	< 0.001
Total	97	440.952	2.966

**Table 7:** Population pairwise *F*_*ST*_ and significant values (*p* < 0.05)

	DT	TN	KT
**DT**		+	+
**TN**	0.072		+
**KT**	0.182	0.146	

**Fig. 8:**
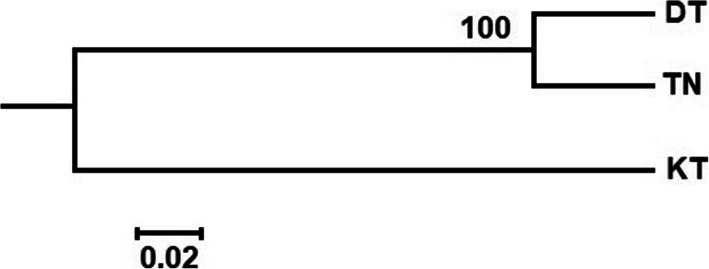
UPGMA dendrogram based on Nei’s chord distance of genetic relationship among three *P. vietnamensis* populations

**Fig. 9:**
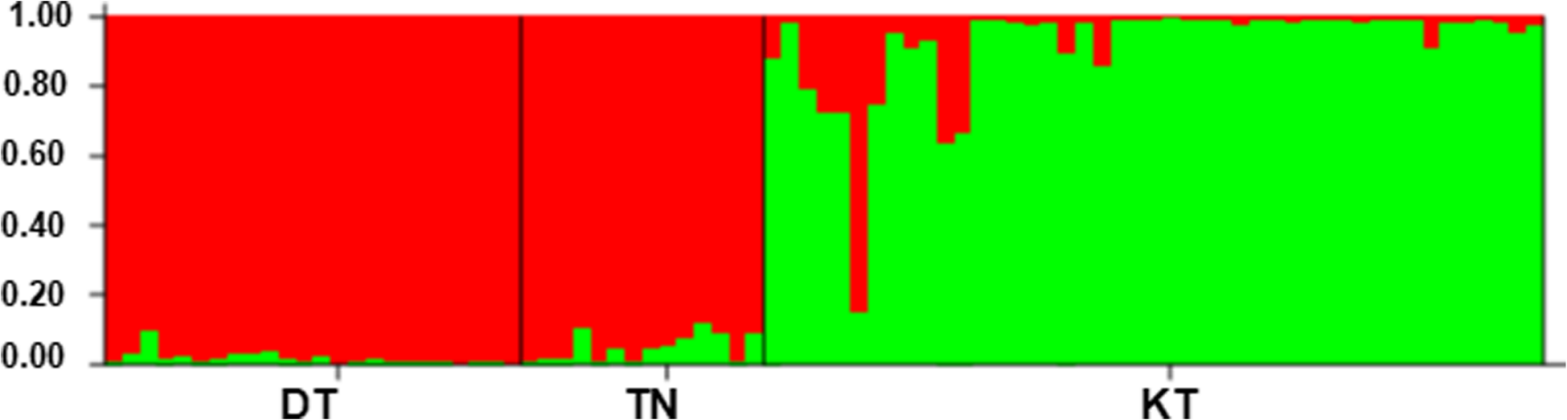
Bar plot of admixture assignment for three *P. vietnamensis* populations to cluster (K = 2) based on Bayesian analysis

The genetic relationship of *P. vietnamensis* populations are expressed in Fig. 8. The DT and TN populations were grouped together and firmed one cluster with the bootstrap value of 100%. In the STRUCTURE analysis, the highest ∆K value (2032.81) (Fig. S1) for 98 individuals revealed K = 2 to be the optimum number of genetic clusters and indicated that all the studied plants exhibited admixture from two clusters. The percentage of ancestry of each population and individuals in two genetic groups shows that one group (red) was predominant in two populations (DT and TN) and second group (green) was predominant in one population i.e. KT (Fig. 9).

## Discussion

Transcriptome sequencing/analysis is very effective tool for gene identification [53–56] and to identify gene expression at different developmental stages or physiological conditions of a cell [47]. Illumina HiSeq™ 4000 technology is effective, timeless, affordable, trusty tool for transcriptome description and gene detection in non-model plants as well. Previous studies showed that the numbers of ESTs were generated from *P. ginseng* leaves [48, 57], *P. notoginseng* roots [58] and American ginseng (*P. quinquefolius* L.) flowers, leaves and roots [44]. To date, many researchers have studied the molecular markers for the genetic analysis of *Panax* species i.e. *P. ginseng* [40–42, 46, 48, 49], *P. notoginseng* [45]. However, due to unavailability of reference genome for *P. vietnamensis*, using Illumina HiSeq™ 4000 the produced reads were assembled through the de novo assembler Trinity. For the first time, we have reported comprehensive transcriptional information for the EST-SSR markers development and then explored the diversity and genetic structure of existent natural *P. vietnamensis* populations.

Illumina paired-end sequencing technology generated 23,741,783 clean and high quality reads with 93.5% Q30 bases and GC content 51.43%. The current results is higher than the previous study [2] on *P. vietnamensis* var. *fuscidiscus* (43.25%), indicating better quality sequencing. In the sequence assembly 89,271 unigenes (average length = 598.32 bp, N50 = 942 bp) were recorded, which was shorter than the results of Cao et al. [47] in *P. ginseng* (average length = 690–698 bp, N50 = 1130–1161 bp) and Zhang et al. [2] in *P. vietnamensis* var. *fuscidiscus* (average length = 1304 bp, N50 = 2018 bp). We had used the same technology; this might be the depth of sequencing, method of assembly and natural features of the plants. The transcriptome sequencing data of *P. vietnamensis* was further explored for genetic diversity, population structure and marker development. The 72,183 (80.86%) unigenes were annotated into 51 GO categories. The “metabolic activities”, “binding” and “cell part” was on the top among biological activities, molecular function and cellular component, respectively. The results are in line with GO functional categories of *P. vietnamensis* var. *fuscidiscus* [2] and *P. ginseng* [47]. The predicted unigenes 5838 (5.42%) through KEGG pathways were mapped into 118 biological pathways and majority of pathways were related to metabolism. The specific pathways, including ribosome, RNA transport spliceosome, purine metabolism and signal transduction of plant hormone etc. These data unveil the active metabolic processes as well as synthesis of multifarious metabolites in the species. In *P. vietnamensis* and other *Panax* species, leaves have high value of aldehydes, esters and terpenoids, these compound help in resistance against biological as well as environmental pressures, such as cold, drought and pests. In the current study, we have recorded the unigenes for signal transduction of the plant hormones that reacts to plant environmental conditions.

Microsatellites are spread in plant genomes and involved in the regulation of their expression and function [13, 59]. The studies on distribution of SSRs in species, the mechanism of SSR variation and comparison are the first step towards elucidation of the function [59, 60]. There are many types of SSR markers and extensively spread in plant genomes [43, 61]. From 8927 unigenes, 11,343 EST-SSR molecular markers were identified by RNA sequencing, while 2763 unigenes have EST-SSR locus more than one. Zhang et al. [2] also identified 21,320 SSRs in *P. vietnamensis* var. *fuscidiscus* with 2918 containing more than one SSR. In the previous studies of Um et al. [49] on *P. ginseng* and Zhang et al. [2] on *P. vietnamensis* var. *fuscidiscus* in EST-SSRs di-nucleotide repeats (60.1 and 52.25%, respectively) were the most abundant type. We have identified SSR markers (11,343) of *P. vietnamensis*, the mono-nucleotide (5004; 44.12%), di-nucleotide (4648, 40.98%) and tri-nucleotide (1563, 13.78%) were the top repeats. The leading was di-nucleotide, tri-nucleotide, tetra-nucleotide repeat motif in *P. vietnamensis* with AG/TC (90.06%), CCG/GGC (22.65%), ACTG/TGAC (19.30%), respectively. Which confirmed the study of Um et al. [49] on *P. ginseng*, but tri-nucleotide repeat motif was different in *P. vietnamensis* than other plants, such as *Myricarubra* [27], *Salix*, *Populus* and *Eucalyptus* [29]. The CG/CG motif (0.17%) was irregularly detected in *P. vietnamensis*, as also observed by Wu et al. [44], which confirmed that the repeat motif CG/CG is not common in numerous dicotyledon plants [62–65].

Genetic diversity has important character in the germplasm improvement and generally used in various medicinal plants [65–68]. The genetic diversity degree in many plants can be linked with the numbers of loci and populations [31, 69], the geographical range size [70] and genetic exchange [71]. In the current research, the nine SSR markers showed a high degree of genetic diversity in *P. vietnamensis* populations and expected heterozygosity (H_O_ = 0.422 and H_E_ = 0.479) compared to some *Panax* species, such as *P. stipuleanatus* [50], *P. ginseng* [72, 73] However, our results are in line with studies of Reunova et al. [46] on *P. ginseng*, the natural species of Russia (H_O_ = 0.453 and H_E_ = 0.393), Liu et al. [74] on *P. notoginseng* (H_E_ = 0.350) and Reunova et al. [75] on *P. vietnamensis* (H_E_ = 0.55) using microsatellite markers. High levels of genetic diversity in three *P. vietnamensis* populations, TN, DT and KT indicated that this species is predominantly outcrossed. High gene flow (N_m_ > 1) may be a consequence of high outcrossing rates in the three populations. Dispersal of pollen grains by insects might be considered as a major factor for this species. Positive fixation index values were detected in all *P. vietnamensis* populations and showed a deficit of heterozygosity due to inbreeding. This suggests small neighborhood size and mattings between siblings within populations. Our results also showed a sign of the bottleneck in all three studied populations (*p* < 0.005). Significant heterozygosity deficits were detected in three populations (TN, DT and KT) under TPM and SMM (*p* < 0.005) models. The models suggested reduction in population size of the targeted populations.

*F*_*ST*_ is trenchant approach for measurement of gene flow in populations and genetic variation [76]. The genetic variation among *P. vietnamensis* populations was moderate. The low *F*_*ST*_ value between two populations (DT and TN) can facilitate strong gene flow within populations (*N*_*m*_ = 3.2). However, the low level of genetic variation between two populations, TN and DT (*F*_*ST*_ = 0.072) might be due to geographical distance. These two populations are located in the same province of Quang Nam. The results of AMOVA analysis also indicated that 63.17% of variation was distributed within individuals and 12.45% among individuals within populations. These results showed a moderate genetic structure of *P. vietnamensis*. Genetic variation among populations is highly affected by genetic drift, gene flow, mutations, selection and long-term evolution [77]. Long lived and outcrossing species maintain high degree of genetic variation in populations and low genetic differentiation in populations, reflecting maximum levels of gene flow. Previous studies reported low differentiation between populations, and reflecting strong gene flow in *P. ginseng* [46, 72] and *P. stipuleanatus* [50]. The strong gene flow among populations might be due to high outcross rates within populations. Thus, pollen grains dispersion through insects can be considered as a major factor of the population structure. The Bayesian analysis and UPGMA tree showed two different groups of genetically mixed individuals of *P. vietnamensis*. In the current study we had isolated population among province through geographical distance. Two populations DT and TN closed together within the province (Quang Nam) and formed a genetic cluster while the KT population in Kon Tum province was separated and formed one cluster by the geographical distance, where gene exchange between the two groups was restricted. The *P. vietnamensis* faced serious threats in their survival. Based on our studied results, all the studied populations can be considered for both in-situ and ex-situ conservation strategies.

## Conclusions

De-novo transcriptome sequencing of *P. vietnamensis* was performed by the Illumina sequencing platform. We produced a large number of ESTs and identified candidate genes that differentially expressed in *P. vietnamensis*. A total of 11,343 EST-SSRs was identified. It is obvious from the data that the natural populations of *P. vietnamensis* maintained high level of genetic diversity. Numerous SSR markers were identified and will contribute to marker-assisted breeding of *P. vietnamensis*. This study does not only provide ground for *P. vietnamensis* breeding but also a platform for its conservation, to maintain genetic diversity.

## Methods

### Plant material

**Fig. 10:**
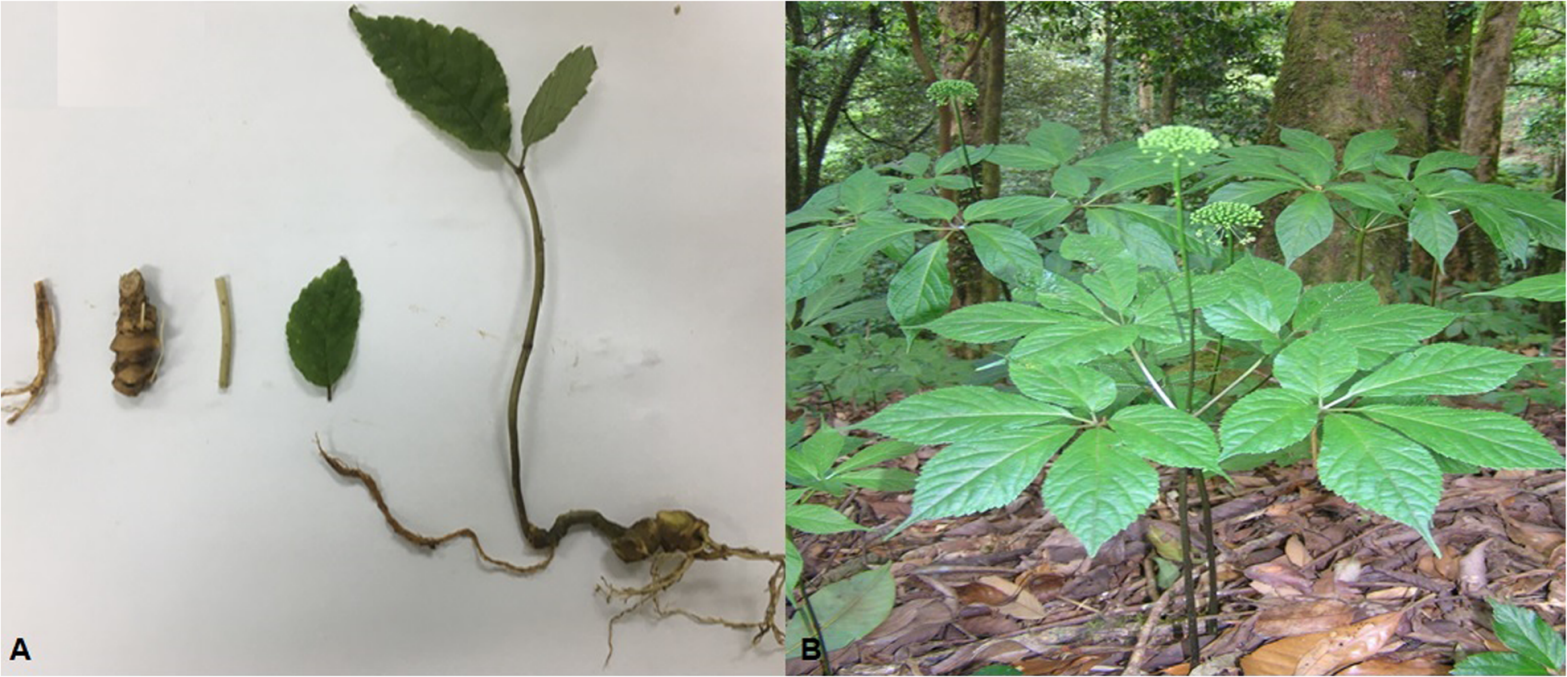
The Leaves, Stem, Roots (**a**) and adult Plant (**b**) of *P. vietnamensis* in Quang Nam province, Vietnam. Photographs by Dinh Duy Vu

We had collected samples (roots, leaves and stems) in liquid nitrogen of *P. vietnamensis* (Fig. 10a) from a wild population (Quang Nam province) for RNA extraction stored at − 80 °C. *P. vietnamensis* (ten plants) wild population (Quang Nam province) was used for EST-SSRs development to test the amplification relevancy of the synthesized EST-SSR primers (Table 8). Three different wild populations (98 Plants) of *P. vietnamensis* were sampled to assess structure and genetic diversity (Fig. 10b, Table 8). The wild population of *P. vietnamensis* was sampled during spring and summer 2019, respectively. For DNA extraction fresh leaves were desiccated in silica gel. This species was identified by Dr. Nguyen Thi Phuong Trang as *Panax vietnamensis* Ha et Grushv (percent identify: 100%) based on the morphology characteristics, and it was further confirmed by the sequence data of the nuclear gene (*ITS-rDNA*) with Genbank accession number MH238443. The permission for samples collection in Quang Nam and Kon Tum provinces (Letter No.123/QĐ-STTNSV dated February 20, 2019 and Letter No. 819/QĐ-STTNSV dated May 10, 2019) were granted by Institute of Ecology and Biological Resources (IEBR) and further confirmed from people committee of Quang Nam and Kon Tum provinces. The voucher specimens of this species were saved in Dept. of Molecular Systematics and Conservation Genetics, Institute of Ecology and Biological Resources (IEBR), Vietnam Academy of Science and Technology.

**Table 8:** Sampling location *P. vietnamensis* from Vietnam in the present study

Population code	Location	Latitude (N)	Longitude (E)	Altitude (m)	Sample size
**TN**	Tra Linh, Nam Tra My, Quang Nam province	15^o^ 1′51.92”	107^o^58’46.44”	1920	18
**DT**	Tak Ngo, Quang Nam province	15^o^ 00′60.7”	108^o^01’66.0”	1567	32
**KT**	Mang Ri, Tu Mo Rong, Kon Tum province	14^o^ 59′11.12”	107^o^57’10.87”	1880	48

### RNA extraction

Total RNA was extracted from each sample (roots, leaves and stems) by the OmniPlant RNA Kit (DNase I) for Illumina sequencing. RNA quality and quantity were validated by Nanodrop and 1.2% agarose gel electrophoresis [18]. Total RNA (equal amount of each sample) was pooled together and sent to Breeding Biotechnologies Co., Ltd., for transcriptome sequencing using Illumina HiSeq™ 4000.

### Transcriptome sequencing and De novo assembly

Cleaned mRNA was used for cDNA library construction extracted from 200 μg of total RNA using Oligo (dT). The cDNA 1st strand was prepared from random hexamers using mRNA as a template and the other strand was from buffer, dNTPs, RNase H and DNA polymerase I, and then cleaned with AMPure XP beads. The cleaned double-stranded cDNA was subjected to terminal repair, the sequencing linker was ligated and then the fragment size was selected with AMPure XP beads. The cDNA library was acquired by PCR enrichment. After library validation on a BioAnalyzer (Agilent 2100), Breeding Biotechnologies Co., Ltd. sequenced the cDNA libraries on a MiSeq (Illumina HiSeq™ 4000).

The Trimmomatic v3.0 [78] was used for raw reads filtration. The reads showing adaptor contamination, length < 36 bp and low quality value (quality < 20) higher than 15% were eliminated. Trinity [79] with default parameters were used for de novo assembly of the cleaned reads. Then the TIGR Gene Indices clustering tool (TGICL) v2.1 [80] was used to cluster and eradicate redundant transcripts, and identified unigenes for further analysis.

### Annotation and functional classification

For functional annotation, all unigene sequences were compared with NCBI non-redundant (NR) protein sequences [81], Swiss-Prot [82], Gene Ontology (GO) [83], Clusters of Orthologous Groups (COG) [84], KOG [85] and KEGG [86] databases using BLAST software [87] to predict the amino acids. The sequence was then aligned using the HMMER software [88] with the Protein family (Pfam) [89] database to obtain Unigene annotation information.

### Development, detection of EST-SSR markers and primer design

The assembled *P. vietnamensis* transcriptome was mined by MISA (Microsatellite identification tool) for markers. The candidate SSRs from 2 to 6 nucleotides range were defined as for dinucleotides, 6 repeats and for all higher order motifs, 5 repeats, according to Jurka and Pethiyagoda [90]. The end-to-end EST-SSRs (interruptions < 100 bp) were considered as compound EST-SSRs. Different nucleotide repeats distribution within UTRs and ORFs in unigenes were analyzed. The annotated SSR-rich unigenes, GO analysis was performed to evaluate its significance. Primer 5.0 [91] at default settings was used for ETS-SSR primers designing to generate PCR products in 100–300 bp size. The primer length was 18–24 bp with an optimum of 20 bp, annealing temperature between 55 and 65 °C with an optimum of 60 °C. A polymorphic maximization criterion was used for the selection of polymorphic loci. For polymorphism maximization, for dinucleotides, trinucleotides and tetranucleotides, SSR loci with minimum ten, seven and five repeats were selected, respectively. Reactions were executed in 25 μl volume, 2 μl of genomic DNA (10 ng total), 12.5 μl Master Mix 2X, 1 μl of each 10 μM primer and 9.5 μl H_2_O deionized. The cycling conditions were 95 °C for 3 min, then 35 cycles of 94 °C for 45 s, 60 °C for 45 s, 72 °C for 45 s, and 72 °C for 10 min at final extension. The PCR products were separated, determined and analyzed according to Vu et al. [18].

### Population genetic analysis

To evaluate population structure and genetic diversity of *P. vietnamensis* precisely, 101 polymorphic SSR markers were carefully chosen and 20 primer pairs were successfully amplified for DNA fragments. Among the selected SSR markers, nine markers had clear and reproducible profiles, and were selected for study (Table 2). DNA isolation kit (Norgenbiotek, Canada) was used for genomic DNA extraction. The samples were crushed by Mixer mill MM 400 (Retsch) in liquid nitrogen. DNA quality and concentration were validated according to Vu et al. [18]. The concentration was then diluted to 10 ng/μl.

PCR was executed in 25 μl volume including 2 μl of genomic DNA (total 10 ng), 12.5 μl Master Mix 2X, 1 μl of each 10 μM primer, and 9.5 μl H_2_O deionized. The reaction was amplified in the thermal cycler conditions: an initial denaturing at 94 °C for 3 min, 40 cycles for 1 min at 94 °C, 30s at 54-56 °C annealing temperature for primer pair (each) and 1 min extension at 72 °C and 10 min at 72 °C for final cycle before holding the samples at 4 °C till analysis. The Sequi-Gen®GT DNA electrophoresis system were used for amplification products separation with 8% polyacrylamide gels in 1 x TAE buffer and then visualized by GelRed™ Nucleic Acid Gel Stain. Alleles size was detected by Gel-Analyzer software of GenoSens 1850 (Clinx Science Instruments Co., Ltd) with a 20 bp DNA ladder (Invitrogen).

Genetic parameters were analyzed on the GenAlEx [92], with the proportion of polymorphic loci (*P*), effective alleles (*A*_*E*_), the number of alleles per locus (*A*). Observed heterozygosities (*H*_*O*_), expected heterozygosities (*H*_*E*_), the fixation index (*F*), the differentiation index between pairwise populations (*F*_*ST*_), the matrix of *F*_*ST*_ between various populations and gene flow (*Nm*) was calculated by the formula *Nm* = [(1/Fst) - 1]/4 [93] Polymorphism information content (PIC) value was calculated according to Botstein et al. [94] Tests for deviation from Hardy-Weinberg equilibrium at the locus (each) and disequilibrium in the linkage for each locus pairwise combination in each population were performed by Genepop v.4.6 [95]. Testing of recent bottleneck events for population (each) via the SSM and TPM were tested using Botteneck v.1.2 [96]. The significance of these tests was measured by the one-tailed Wilcoxon signed rank test. The proportion of the SSM was set to 70% under default settings. The genetic distances among populations were also calculated using GenAlEx. The significance of F_ST_ values in each population pair across all loci was tested by applying the sequential Bonferroni correction.

The data were subjected to AMOVA using Arlequin 3.1 and significance test was applied on a basis of 10,000 permutations [97]. The UPGMA approach was used to determine the genetic association among population by Poptree2 [98]. STRUCTUREv.2.3.4 was used to explore population structure with Bayesian clustering approach [99]. The admixture model was set with correlated allele frequencies i.e. in the data set (K), in different groups, ten separate runs were employed for K within 1 and 15 at 500,000 Markov Chain Monte Carlo (MCMC) repetitions and at 100,000 burn–in the period. Structure Harvester [100] was used for the group (number) detection that best fits in the dataset based on the ∆K according to Evanno et al. [101].

## Supplementary information

**Additional file 1: Figure S1.** The Delta K distribution graph.

## Data Availability

The data charts supporting the results and conclusions are included in the article and additional files. Partial cds the SSR sequences data have been deposited inthe NCBI under accession number from MK802095 to MK802103 (https://www.ncbi.nlm.nih.gov/).

## Abbreviations

AMOVA: Analysis of molecular variance
COG: Clusters of orthologous groups
EST-SSRs: Expressed sequence tag-simple sequence repeat
GO: Gene ontology categories
KEGG: Kyoto encyclopedia of genes and genomes pathways
UPGMA: Unweighted pair group method of arithmetic average
SSM: Stepwise mutation model; TPM: two phase model
