# Supplementary figures and images for: De novo assembly and Transcriptome characterization of an endemic species of Vietnam, Panax vietnamensis Ha et Grushv., including the development of EST-SSR markers for population genetics
Source: BMC Plant Biol. 2020 Jul 29;20:358. doi: 10.1186/s12870-020-02571-5 (PMC7391578; doi:10.1186/s12870-020-02571-5)

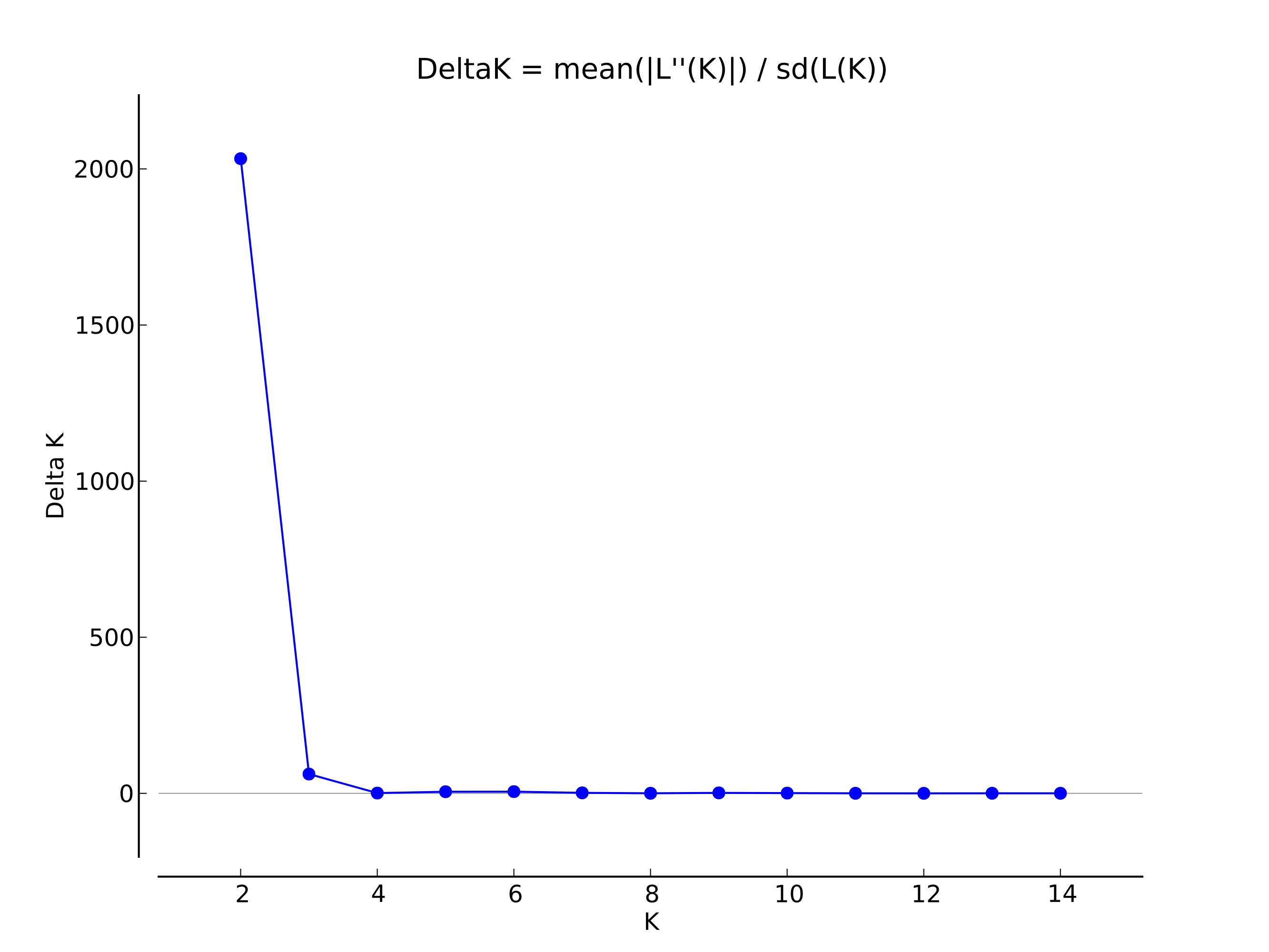

Supplement: Supplementary file 1 — Additional file 1: Figure S1. The Delta K distribution graph. [file 12870_2020_2571_MOESM1_ESM.png]
